# Supplementary material for: Artificial Extracellular Matrices Containing Bioactive Glass Nanoparticles Promote Osteogenic Differentiation in Human Mesenchymal Stem Cells
Source: Int J Mol Sci. 2021 Nov 26;22(23):12819. doi: 10.3390/ijms222312819 (PMC8657909; doi:10.3390/ijms222312819)
Supplement: Supplementary file 1 [file ijms-22-12819-s001.zip › ijms-1471042-supplementary.pdf]

Supplementary data

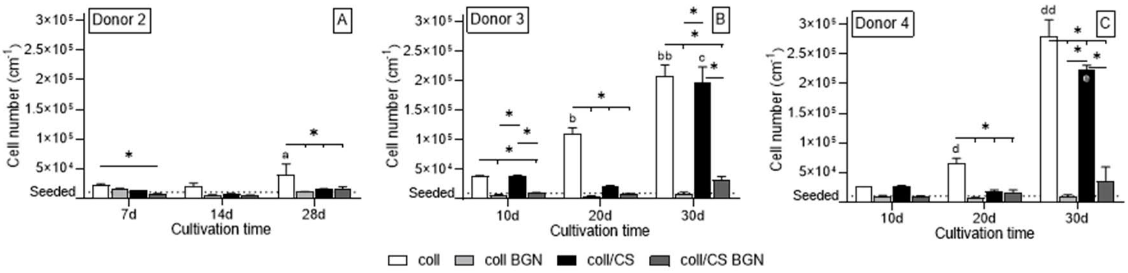

Figure S1: Cell proliferation. Cellular growth of hMSC as determined by analysis of the DNA content after addition of differentiation supplements. A. hMSC on aECM coatings without dexamethasone (Dex; donor 2) and B/C. hMSC on aECM coatings with Dex (donor 3/4); n = 4. Statistics: 2-way ANOVA $^*p < 0.05$ was considered as significant. Additionally, a statistical difference for the following data was found: statistical significant against: a – 7d and 14d coll; b – 10d coll; bb – 10d and 20d coll; c – 10d and 20d coll/CS; d – 10d coll; dd – 10d and 20d coll; e – 10d and 20d coll/CS.

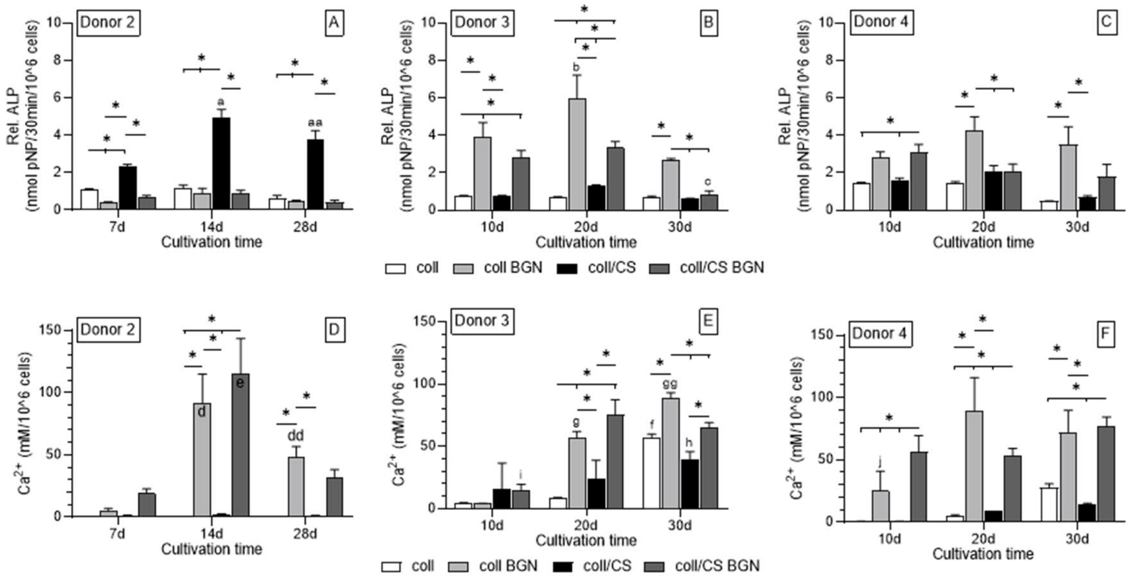

Figure S2: ALP activity and calcium deposition. Relative ALP activity (A-C) and calcium deposition (D-F) of hMSC after addition of differentiation supplements. hMSC on aECM coatings w/o Dex (Donor 2) and coatings with Dex (Donor 3/4); n = 4. Statistic: in 2-way ANOVA p-values $^*p < 0.05$ were considered significant. Additionally, a statistical difference were found to: a – 7d coll/CS; aa – 7d and 14d coll/CS; b – 10d and 30d coll BGN; c – 10d and 20d coll/CS BGN; d – 7d coll BGN; dd – 7d and 14d coll BGN; e – 7d and 28d coll/CS BGN; f – 10d and 20d coll; g – 10d coll BGN; gg – 10d and 20d coll BGN; h – 10d coll/CS; i – 20d and 30d coll/CS BGN; j – 20d and 30d coll BGN.

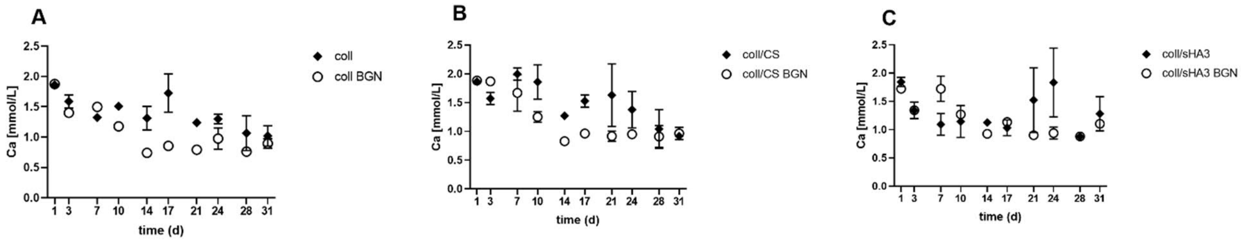

Figure S3: Calcium concentration in cell culture supernatants, measured by ICP-OES.
